# RNA Sequence Reveals Mouse Retinal Transcriptome Changes Early after Axonal Injury

**DOI:** 10.1371/journal.pone.0093258

**Published:** 2014-03-27

**Authors:** Masayuki Yasuda, Yuji Tanaka, Morin Ryu, Satoru Tsuda, Toru Nakazawa

**Affiliations:** Department of Ophthalmology, Tohoku University Graduate School of Medicine, Sendai, Japan; University of Rochester, United States of America

## Abstract

Glaucoma is an ocular disease characterized by progressive retinal ganglion cell (RGC) death caused by axonal injury. However, the underlying mechanisms involved in RGC death remain unclear. In this study, we investigated changes in the transcriptome profile following axonal injury in mice (C57BL/6) with RNA sequencing (RNA-seq) technology. The experiment group underwent an optic nerve crush (ONC) procedure to induce axonal injury in the right eye, and the control group underwent a sham procedure. Two days later, we extracted the retinas and performed RNA-seq and a pathway analysis. We identified 177 differentially expressed genes with RNA-seq, notably the endoplasmic reticulum (ER) stress-related genes *Atf3*, *Atf4*, *Atf5*, *Chac1*, *Chop*, *Egr1* and *Trb3*, which were significantly upregulated. The pathway analysis revealed that ATF4 was the most significant upstream regulator. The antioxidative response-related genes *Hmox1* and *Srxn1*, as well as the immune response-related genes *C1qa*, *C1qb* and *C1qc*, were also significantly upregulated. To our knowledge, this is the first reported RNA-seq investigation of the retinal transcriptome and molecular pathways in the early stages after axonal injury. Our results indicated that ER stress plays a key role under these conditions. Furthermore, the antioxidative defense and immune responses occurred concurrently in the early stages after axonal injury. We believe that our study will lead to a better understanding of and insight into the molecular mechanisms underlying RGC death after axonal injury.

## Introduction

Glaucoma is a leading cause of blindness worldwide [1]. It is characterized by glaucomatous optic neuropathy (GON), and is associated with optic nerve degeneration that results in progressive visual dysfunction [2]. In glaucoma patients, the number of retinal ganglion cells (RGCs) decreases due to axonal degeneration, resulting in visual dysfunction. Despite the attempts of many clinicians and scientists to identify the molecular mechanisms of pathogenesis in glaucoma, they are not yet well understood, possibly because of the multifactorial nature of glaucoma [3].

High intraocular pressure (IOP) is widely recognized as a major risk factor for glaucoma, and treatment to lower IOP is currently the only method that evidence has shown to prevent the progression of the disease [4]. Recently, many varieties of IOP-lowering eye drops have become clinically available to treat glaucoma. However, substantial reductions in IOP, up to 30%, fail to halt the progress of visual dysfunction in some patients, particularly those with normal tension glaucoma (NTG) [5]. In addition to IOP, risk factors for NTG include age, myopia [6], parapapillary atrophy (PPA) [7] and reduced ocular blood flow [8]. There is thus a necessity for further investigation of these IOP-independent mechanisms, and the development of new neuroprotective drug targets for glaucoma.

Many recent investigations have led to a growing understanding of the underlying mechanism of RGC death in glaucoma, which previous studies had found to be induced by axonal injury to the lamina cribrosa [3]. However, those studies were mainly designed around approaches that focused on only a few pathways [9], [10]. In order to overcome the heterogeneous and multifactorial nature of glaucoma and find novel critical molecular targets for treatment, it is necessary to use a global approach (i.e., one including the transcriptome and proteome).

A simple animal model mimicking the pathogenesis of glaucoma is a useful tool in investigations of the mechanism of RGC death, because standard excisional biopsy is impossible in the case of the human retina [11]. In one of the most widely used models, optic nerve crush (ONC) is performed in mice to induce axonal injury, which is a contributor to the progression of RGC death in glaucoma [10], [12]–[14]. Interestingly, in this model the number of RGCs is maintained for a short duration after ONC, and significant RGC loss is not observed until day 3 [10]. Significant axonal damage is known to occur in the retina before visual field defects become detectible [15]. It would therefore be very useful to develop diagnostic methods and drug targets that functioned in these early stages of glaucoma. Analysis of post-ONC mouse retinas in the early stages of axonal injury, before RGC loss (i.e., on day 2), may give us valuable insights to help achieve this goal.

Microarray analysis is a common way to evaluate the expression level of large numbers of genes simultaneously. It has also been used to evaluate changes after axonal injury both in the retina in general and in isolated RGCs [16]. However, microarrays are only capable of measuring known transcripts, and do not allow the investigation of total genetic changes. By contrast, RNA sequencing (RNA-seq) is able to assess complete genes and splice variants, with a high degree of reproducibility that matches that of microarrays [17]. RNA-seq technology thus has the potential to give us very useful, detailed information on the mechanisms of disease, as well as unknown pathways and networks of disease, that may lead to the discovery of new treatment strategies.

The purpose of this study was thus to use RNA-seq to investigate the molecular mechanisms of damage in the early stages of the response to axonal injury, before the onset of RGC death. We believe that our study may open new avenues of investigation into treatment strategies for axonal damage associated with ocular diseases, especially glaucoma.

## Results

### RNA-seq analysis and global gene expression profiles in axonal injury

**Figure 1:**
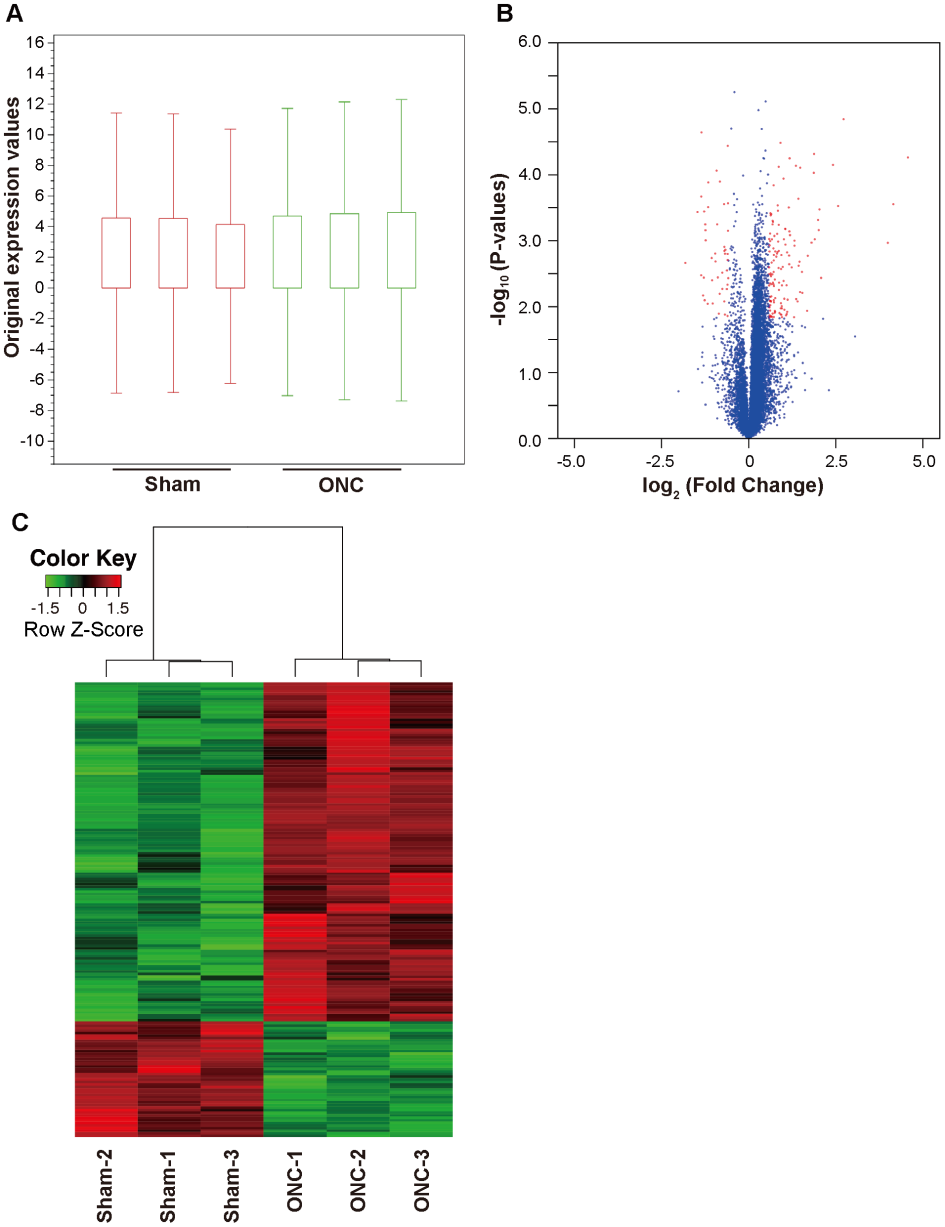
Gene expression profiles of the samples. (A) Box plot showing overall RPKM expression values for the ONC and control samples. (B) Volcano plot showing differentially expressed genes after axonal injury. For each plot, the X-axis represents log_2_ FC and the Y-axis represents -log_10_ (P-values). DEGs are shown as red dots. (C) Hierarchical clustering of DEGs after ONC. Red indicates increased expression and green indicates decreased expression. DEGs were defined as having absolute FC > 1.5 and a FDR < 0.1.

In order to investigate the transcriptome profile at an early stage after axonal injury, but before significant RGC loss [10], we performed an RNA-seq analysis of mouse retinas harvested 2 days after ONC or sham operations. In order to obtain triplicate results, three samples were obtained from each group, each sample being a combination of material from six unique retinas. Each of these samples was sequenced on one lane of the Illumina HiSeq2000 platform (Illumina, San Diego, CA). All sequence reads were mapped to the reference genome (NCBI37/mm9) with CLC Genomics Workbench (version 6.0.1) (CLC Bio, Aarhus, Denmark) [18], [19]. The total number of reads per lane was approximately 400 million, and the total number of reads per sample ranged from 62.9 to 70.3 million. An average 73.8% of total reads were mapped in pairs to the reference genome (Table S1). Detailed mapping statistics are listed on Table S2. To determine the expression level of various genes and compare them between samples, we used variable RPKM (reads per kilobase of exon per million mapped reads) [20]. To examine the overall distribution of gene expression values, we created box plots of RPKM expression values with CLC Genomics Workbench (Figure 1A). Overall RPKM expression values were similar in each sample. We excluded genes that did not have a mean RPKM > 0.3 in at least one group, in order to remove background noise [21]. The number of genes with a mean RPKM > 0.3 in at least one group was 13160. These were used for the differential gene expression analysis [19]. Fold change (FC) differences between the mice that underwent ONC and those that underwent a sham operation were calculated. The Student’s t-test was performed to compare the groups with R software (version 3.0.1) [22]. *P*-values were adjusted for multiplicity with the Bioconductor package qvalue to control the false discovery rate (FDR) [23]. Differentially expressed genes (DEGs) were defined as those with |FC| > 1.5 and FDR < 0.1 [24]. It is known that the cells affected by ONC are mainly RGCs. The abundance ratio of RGCs in retinal tissue has been reported to very low (less than 0.5%) [25]. Furthermore, an approximately 1.5 to 2-fold increase in gene expression (e.g., in *Jun, Jund and Gadd45a)* has been reported to be a significant change in a previous analysis of changes in the entire retina after optic nerve crush [26]. We therefore applied this relatively lower cutoff (|FC| > 1.5 and FDR < 0.1) in the current study. We created a volcano plot showing DEGs as red dots with the ggplot2 package in R software [27] (Figure 1B). We also conducted a hierarchical clustering analysis of DEGs from all samples with Ward’s method of Euclidean distances [28], and created a heatmap with the heatmap.2 function of the gplots package of R software [29]. The results indicated that gene expression was similar in each group (Figure 1C).

### Differentially expressed genes after ONC

The triplicate samples from the ONC and sham groups were assayed for DEGs, and 177 DEGs (132 up- and 45 downregulated genes) were identified (Table S3). The 10 most up- and downregulated genes are listed in Table 1. The expression changes of known RGC markers and axon regeneration markers [16], [30], [31] are summarized in Table 2. We found that the following RGC markers were significantly downregulated 2 days after ONC: *Nefh* (–2.24-fold), *Pou4f1* (–1.54-fold), *Pou4f2* (–2.40-fold), *Rbpms* (–1.62-fold) and *Sncg* (–1.77-fold). Interestingly, the expression of *Thy1* and *Pou4f3* did not change significantly at this time point (day 2). Generally, the expression of *Thy1* begins to decrease 3 days after axotomy in rats [32]. The following axon regeneration markers were significantly upregulated after ONC: *Gap43* (1.53-fold) and *Sprr1a* (23.81-fold).

**Table 1:** Top 10 upregulated and downregulated genes after ONC.

Symbol	Description	Gene accession	Fold change	*P*-value	FDR
**Upregulated**					
*Sprr1a*	Small proline-rich protein 1A	NM_009264	23.81	5.46E-05	0.026
*Mmp12*	Matrix metallopeptidase 12	NM_008605	17.82	2.80E-04	0.045
*Ecel1*	Endothelin converting enzyme-like 1	NM_021306	15.96	1.08E-03	0.054
*Chac1*	ChaC, cation transport regulator-like 1 (E. coli)	NM_026929	6.61	1.44E-05	0.022
*Sox11*	SRY-box containing gene 11	NM_009234	5.92	2.98E-04	0.045
*Atf3*	Activating transcription factor 3	NM_007498	5.34	7.10E-05	0.028
*Lgals3*	Lectin, galactose binding, soluble 3	NM_001145953	4.23	3.66E-03	0.071
*Phgdh*	3-phosphoglycerate dehydrogenase	NM_016966	4.09	3.36E-04	0.045
*Cdkn1a*	Cyclin-dependent kinase inhibitor 1A (P21)	NM_007669	4.03	6.92E-04	0.051
*Tnfrsf12a*	Tumor necrosis factor receptor superfamily, member 12a	NM_013749	3.98	4.84E-04	0.048
**Downregulated**					
*Gm6747*	Predicted gene 6747	XM_003945591	–3.53	2.17E-03	0.061
*Irx2*	Iroquois related homeobox 2 (Drosophila)	NM_010574	–2.77	3.69E-04	0.045
*Gm7244*	Predicted gene 7244	NG_019018	–2.57	3.35E-03	0.069
*Rasgrp2*	RAS, guanyl releasing protein 2	NM_011242	–2.57	2.13E-04	0.043
*Tppp3*	Tubulin polymerization-promoting protein family member 3	NM_026481	–2.55	2.27E-05	0.022
*Kcnd2*	Potassium voltage-gated channel, Shal-related family, member 2	NM_019697	–2.47	6.78E-03	0.081
*Opn3*	Opsin 3	NM_010098	–2.44	3.63E-03	0.071
*Ctxn3*	Cortexin 3	NM_001134697	–2.42	3.62E-04	0.045
*Pou4f2*	POU domain, class 4, transcription factor 2	NM_138944	–2.40	7.99E-03	0.085
*Pvalb*	Parvalbumin	NM_013645	–2.37	5.66E-04	0.048

Differences were considered significant when FDR was < 0.1 and |FC| was > 1.5.

**Table 2:** Expression changes in genes associated with RGCs, axon regeneration and ER stress after ONC.

Symbol	Description		Gene accession	Fold change	*P*-value	FDR
**RGC**						
*Nefh*	Neurofilament, heavy polypeptide		NM_010904	–2.24	3.11E-04	0.045
*Pou4f1*	POU domain, class 4, transcription factor 1		NM_011143	–1.54	5.41E-03	0.077
*Pou4f2*	POU domain, class 4, transcription factor 2		NM_138944	–2.40	7.99E-03	0.085
*Pou4f3*	POU domain, class 4, transcription factor 3		NM_138945	1.04	NS	NS
*Rbpms*	RNA binding protein gene with multiple splicing		NM_019733	–1.62	1.43E-03	0.056
*Sncg*	Synuclein, gamma		NM_011430	–1.77	1.27E-04	0.032
*Thy1*	Thymus cell antigen 1, theta		NM_009382	–1.07	NS	NS
**Axon regeneration**						
*Gap43*	Growth associated protein 43		NM_008083	1.53	4.44E-03	0.073
*Sprr1a*	Small proline-rich protein 1A		NM_009264	23.81	5.46E-05	0.026
**ER stress**						
*Atf3*	Activating transcription factor 3		NM_007498	5.34	7.10E-05	0.028
*Atf4*	Activating transcription factor 4		NM_009716	1.61	5.65E-04	0.048
*Atf5*	Activating transcription factor 5		NM_030693	2.24	2.27E-03	0.062
*Chac1*	ChaC, cation transport regulator-like 1 (E. coli)		NM_026929	6.61	1.44E-05	0.022
*Ddit3*	DNA-damage inducible transcript 3		NM_007837	2.15	1.51E-03	0.056
*Egr1*	Early growth response 1		NM_007913	2.25	7.25E-04	0.051
*Trib3*	Tribbles homolog 3 (Drosophila)		NM_175093	2.89	2.98E-03	0.067

Differences were considered significant when FDR was < 0.1 and |FC| was > 1.5. NS  =  not significant.

A review of the published literature revealed that the following sets of endoplasmic reticulum (ER) stress-related genes have been shown to be significantly upregulated 2 days after ONC: *Atf3*, *Atf4*, *Atf5*, *Chac1*, *Ddit3*, *Egr1*, *Trib3* [33]–[35] (Table 2).

### Significant networks and biological functions after ONC revealed by pathway analysis

**Figure 2:**
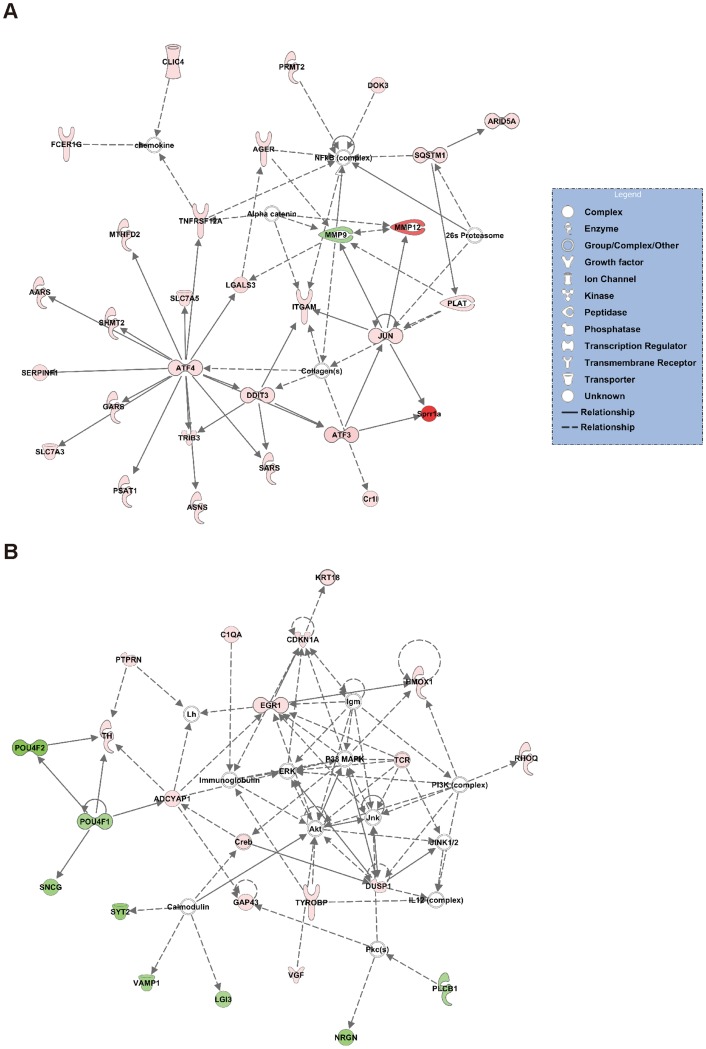
Network analysis of the effect of ONC on gene expression. These post-ONC significance networks were generated by IPA. The 2 most significant networks are shown. (A) Network 1 was associated with the “Cell Death and Survival”, “Cancer” and “Cell Morphology” pathways. (B) Network 2 was associated with the “Neurological Disease”, “Nervous System Development and Function” and “Tissue Morphology” pathways. Red indicates upregulated genes, green indicates downregulated genes, and white indicates genes that were not annotated in this RNA-seq result but that formed part of the network.

To investigate the pathways involved in axonal injury, the DEG dataset was uploaded to Ingenuity Pathway Analysis (IPA, Ingenuity Systems, Redwood City, CA) and mapped to the Ingenuity Pathways Knowledge Base (IPKB) [36]. The 2 most significant networks are shown in Figure 2. Network 1 (Figure 2A) was associated with the “Cell Death and Survival”, “Cancer” and “Cell Morphology” pathways. Network 2 (Figure 2B) was associated with the “Neurological Disease”, “Nervous System Development and Function” and “Tissue Morphology” pathways. Table 3 lists the 5 most significant molecular and cellular functions. The most significant biofunction response, according to IPA, was for the “Cell Death and Survival” pathway, which involved 45 genes (Table S4).

**Table 3:** Top 5 molecular and cellular functions significantly modulated after ONC.

Category	*P*-value	Number of Molecules
Cell Death and Survival	7.45E-07-1.83E-02	45
Cellular Function and Maintenance	2.81E-06-1.83E-02	41
Cell-To-Cell Signaling and Interaction	4E-06-1.83E-02	40
Molecular Transport	5.13E-05-1.17E-02	42
Small Molecule Biochemistry	5.13E-05-1.83E-02	36

Significances were calculated with Fisher’s exact test.

Differences were considered significant at the *P* < 0.05 level.

### RT-PCR validation of RNA-seq data

To validate the RNA-seq findings, we prepared new mouse retinas in each group, and performed RT-PCR on these new groups of retinas. We selected 14 genes (*Sprr1a, Mmp12*, *Sox11*, *Atf3*, *Tnfrsf12a*, *Hmox1*, *Plat*, *Egr1*, *Atf5*, *Ddit3*, *Jun*, *Pou4f2*, *Nefh* and *Pou4f1*) involved in the “Cell Death and Survival” pathway, and examined changes in their expression with RT-PCR (Table 4). We found that results obtained with RT-PCR were similar to those obtained with RNA-seq.

**Table 4:** RT-PCR validation of the expression of selected genes related to the “Cell Death and Survival” pathway.

Symbol	Description	Gene Accession	RNA-seq		RT-PCR	
			FC	*P*-value	FC	*P*-value
*Sprr1a*	Small proline-rich protein 1A	NM_009264	23.81	5.46E-05	232.12	2.79E-03
*Mmp12*	Matrix metallopeptidase 12	NM_008605	17.82	2.80E-04	84.75	2.27E-04
*Sox11*	SRY-box containing gene 11	NM_009234	5.92	2.98E-04	10.14	3.74E-04
*Atf3*	Activating transcription factor 3	NM_007498	5.34	7.10E-05	5.51	1.13E-02
*Tnfrsf12a*	Tumor necrosis factor receptor superfamily member 12a	NM_001161746	3.98	4.84E-04	7.57	1.27E-03
*Hmox1*	Heme oxygenase (decycling) 1	NM_010442	3.67	4.82E-05	4.50	1.53E-02
*Plat*	Plasminogen activator, tissue	NM_008872	2.26	5.63E-05	2.22	8.82E-03
*Egr1*	Early growth response 1	NM_007913	2.25	7.25E-04	4.21	2.15E-02
*Atf5*	Activating transcription factor 5	NM_030693	2.24	2.27E-03	2.82	5.50E-05
*Ddit3*	DNA-damage inducible transcript 3	NM_007837	2.15	1.51E-03	2.37	8.60E-06
*Jun*	Jun oncogene	NM_010591	2.00	1.14E-04	2.22	1.78E-03
*Pou4f2*	POU domain, class 4, transcription factor 2	NM_138944	–2.40	7.99E-03	–2.55	2.34E-06
*Nefh*	Neurofilament, heavy polypeptide	NM_010904	–2.24	3.11E-04	–2.36	3.58E-07
*Pou4f1*	POU domain, class 4, transcription factor 1	NM_011143	–1.54	5.41E-03	–2.25	1.55E-03

Differences between the NC and sham groups were analyzed with the t-test (RT-PCR: n  =  6 for each group). Differences were considered significant at the *P*<0.05 level.

### Upstream analysis and global network interactions after axonal injury

**Figure 3:**
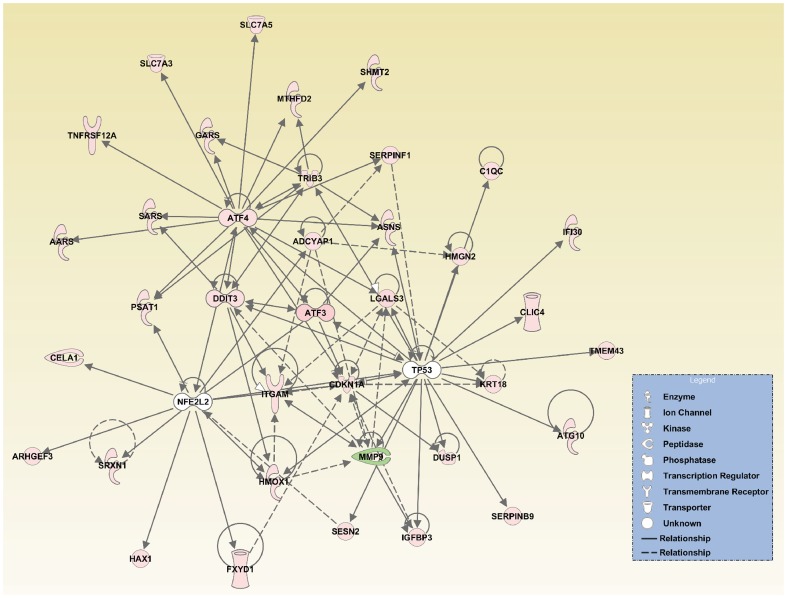
Interaction networks involved in axonal injury after ONC. The upstream analysis was performed with IPA. ATF4, TP53, NFE2L2, DDIT3 and the target molecules of these upstream regulators were merged for this representation of the interaction networks after ONC.

In order to investigate molecular network interactions, IPA performed an upstream regulator analysis. Table 5 shows the transcription factors that IPA predicted to be upstream regulators. The most significant was ATF4, but TP53, nuclear factor (erythroid-derived 2)-like 2 (NFE2L2) and DNA-damage inducible transcript 3 (DDIT3) were also determined to be upstream regulators activated after ONC. Data for ATF4, TP53, NFE2L2, DDIT3 and the target genes from the dataset were merged to create a graphical representation of the network of molecular relationships following ONC (Figure 3).

**Table 5:** Predicted upstream regulators belonging to transcription factors after ONC.

Name	Predicted activation	Activation Z-score	*P*-value of overlap	Target molecules in dataset
ATF4	Activated	3.12	8.90E-18	AARS, ASNS, ATF3, CDKN1A, DDIT3, GARS, LGALS3, MTHFD2, PSAT1, SARS, SERPINF1, SHMT2, SLC7A3, SLC7A5, TNFRSF12A, TRIB3
TP53	Activated	2.44	2.83E-05	ATF3, ATG10, C1QC, CDKN1A, CLIC4, DUSP1, HMGN2, HMOX1, IFI30, IGFBP3, KRT18, LGALS3, MMP9, SERPINB9, SESN2, TMEM43, TRIB3
NFE2L2	Activated	2.13	4.79E-03	ADCYAP1, ARHGEF3, CELA1, FXYD1, HAX1, HMOX1, PSAT1, SRXN1
DDIT3	Activated	2.00	3.20E-06	ATF3, ITGAM, SARS, TRIB3

Data were analyzed with Fisher’s exact test. Differences were considered significant with a *P*<0.05 and |Z-score| ≥ 2.

## Discussion

In this study, we used RNA-seq to examine the global transcriptome profile early after axonal injury, before the onset of significant RGC death. We identified 177 DEGs including previously uninvestigated molecules in ONC. A pathway analysis of these DEGs revealed that the most significant biofunction in axonal injury was the “Cell Death and Survival” pathway. We found that the ATF4-regulated pathway and other sets of ER stress-related genes were significantly upregulated, and that NFE2L2 was also involved in axonal injury, as an upstream regulator. These results point to the critical role that ER stress plays in axonal damage-induced RGC death after ONC. Furthermore, the molecular mechanism of the response to axonal injury also involved antioxidative defense.

This study relied on an animal model of ocular disease in which axonal injury was induced by ONC [10]. Many animal models have been used in recent investigations of novel treatment strategies for glaucoma, such as neuroprotection. Various methods of inducing RGC loss in animals have previously been described, including ONC [10], [14], [37], optic nerve axotomy [32], [38], [39], intravitreal administration of N-methyl-d-aspartate [40]–[42] or Kainic acid [43]–[45] induction of glutamate excitotoxicity, and tumor necrosis factor-α-induced neuroinflammation [46], [47]. In contrast to other models, ONC and optic nerve axotomy induce axonal damage by direct optic nerve injury, which is the main pathogenic component leading to RGC death in glaucoma [14]. In models using glutamate toxicity, RGC death occurs immediately with TUNEL signals detectible within 6 hours after injury [48], [49]. On the other hand, in models using neuroinflammation, RGC death takes a few weeks and only a small number of cells are susceptible. Since the number of surviving RGCs did not significantly decrease until 3 days after ONC in mice [10], we were able to examine retinas on the second day after ONC and investigate the transcriptome profile of axonal injury-induced changes before the onset of RGC death. ONC was thus the most appropriate model of glaucoma for our study.

To our knowledge, this is the first report to use RNA-seq analysis to investigate the retinal transcriptome profile early after axonal injury. Although several researchers have conducted microarray analyses of axonal injury [16], [50], the molecular mechanisms remain unclear. In contrast to RNA-seq, expression microarrays have a number of limitations (e.g., reliance on existing knowledge about the genome sequence, background noise and lower dynamic range). We therefore performed RNA-seq to generate a global view of the transcriptome after axonal injury.

Microarray analysis of rodent RGCs isolated with fluorescence-assisted cell sorting (FACS) has already been reported, and clarified the mechanism of axon regeneration after optic nerve axotomy [16]. Our RNA-seq analysis, by contrast, included cells from the entire retina. Since retinal glial cells are also affected by axonal damage after ONC [51], we therefore hypothesized that RNA-seq analysis of the entire retina would yield information that had not been revealed by previous microarray analyses of FACS-purified RGCs.

In axonal injury, RGCs decrease due to retrograde axonal degeneration [10]. Several RGC marker genes are known to be downregulated in response to axonal injury [30], [52]. In the current study, *Pou4f1* (also known as Brn3a) and *Pou4f2* (also known as Brn3b) were downregulated 2 days after ONC (Table 2). Brn3 is a transcriptional factor expressed in the retina [53]. Furthermore, Brn3a is known to be a useful RGC marker, which can be used to identify and quantify RGCs both in controls and injured retinas [30]. In our study, *Thy1*, another well-known RGC marker [54], did not decrease significantly (Table 2). The loss of Brn3a-positive RGCs was detected earlier than the loss of Fluorogold-labeled RGCs [30]. *Brn3a* may therefore be a useful marker for evaluating RGC dysfunction in the early stages after ONC.

Axotomized RGCs are known to show many similar changes in gene expression during axon regeneration [55]. We found that *Sprr1a* and *Gap43*, genes that are related to axon regeneration, were significantly upregulated in the retina after axonal injury (Table 2). These results support previous findings obtained from a microarray analysis of FACS-purified RGCs taken from retinas subjected to axonal injury [16].

ER stress is thought to play an important role in the pathogenesis of several neurological disorders [56]. ER stress activates three unfolded protein pathways (UPRs) including RNA-dependent protein kinase (PKR)-like ER kinase (PERK), inositol-requiring kinase 1 (IRE1) and ATF6. Prolonged ER stress can also induce apoptosis [35]. In the current study, the ER stress-related genes *Atf3*, *Atf4*, *Atf5*, *Chac1*, *Ddit3* (also known as C/EBP homologous protein (CHOP)), *Egr1* and *Trib3* were significantly upregulated 2 days after ONC (Table 2). Furthermore, IPA predicted that ATF4 was the most significant upstream regulator (Table 5). Under ER stress conditions, ATF4 is induced by eukaryotic inactivation factor 2α, downstream of the PERK pathway [57]. This suggests that ATF4 is the key upstream transcription factor induced by ER stress in the early stages of axonal injury.

IPA also predicted that CHOP was a significant upstream regulator (Table 5). CHOP is transactivated by ATF4, leading to ER stress-induced apoptosis [58]. Deletion of CHOP has been found to promote RGC survival [59]. According to IPA, ATF4 was an upstream regulator of CHOP (Table 5). This suggests that the ATF4-CHOP pathway plays an important role in axonal damage-induced RGC death. We also found that *Jun* was significantly upregulated (Table 4). JUN is known to be activated by the IRE1-JNK pathway under ER stress conditions [60], and can induce apoptosis [26]. Furthermore, we found that ER stress related-genes such as *Trib3* and *Chac1* were significantly upregulated after ONC (Table 2). TRIB3 has been reported to be involved in ER stress-induced apoptosis in 293 and Hela cells [35]. CHAC1 is involved in glutathione depletion and ROS generation [61] and is a proapoptotic component of the UPR, downstream of the ATF4-ATF3-CHOP cascade in primary human aortic endothelial cell lines [62]. To our knowledge, the role of TRIB3 and CHAC1 has not yet been investigated in the retina. The multiple ER stress-related pathways discussed above were activated concurrently in the retina after ONC. Therefore, a network-based approach [63], considering multiple pathways and molecules leading to cell death, is likely the best approach to treatment aimed at RGC protection after axonal injury, resembling the approach to photoreceptor protection that targets two cell death pathways [64].

Additionally, oxidative stress has been implicated in many neurodegenerative diseases [65], [66]. In the current study, we found that the antioxidative response-related genes *Hmox1* and *Srxn1* were significantly upregulated 2 days after ONC (Table S3). IPA indicated that NFE2L2 was one of the upstream regulators activated after ONC, and that the increased expression of *Hmox1* and *Srxn1* was a downstream effect of NFE2L2 activation (Table 5 and Figure 3). NFE2L2, also known as Nrf2 (NF-E2 related factor 2), is a potent transcriptional activator and plays a central role in inducing the expression of many cytoprotective genes such as *Hmox1* and *Srxn1* [67], [68]. Its translocation into the nucleus has been observed at an early stage after ONC [9]. This study also revealed that *Cdkn1a* (also known as p21) was significantly upregulated, and indicated that it interacts with Nrf2 (Figure 3). Cytoplasmic p21 has been reported to enhance axonal regeneration and functional recovery after spinal injury in rats [69]. Furthermore, it has been reported that transcriptional activation of cytoprotective genes by Nrf2 is potentiated in the presence of p21 through facilitated stabilization of Nrf2 [70]. In summary, the results of our study indicate that the Nrf2-related pathway is activated in response to axonal injury, which may be involved in a part of the defense mechanism suppressing RGC death and promoting axonal regeneration in the early stages of axonal injury. Enhancement of the antioxidant response, along with the inhibition of ER stress-related pathways (e.g., ATF-CHOP), may have a synergistic protective effect against RGC death after axonal injury.

The immune response has been reported to be involved in central nervous system (CNS) injury [55]. Our study found that *C1qa*, *C1qb* and *C1qc*, components of C1q belonging to the classical complement pathway, were significantly upregulated (Table S3). Furthermore, *C1qa* was included in the “Cell Death and Survival” pathway according to IPA (Table S4). C1q has been reported to be implicated in the pathogenesis of neurodegenerative diseases such as Alzheimer’s disease [71]. A previous study used a microarray analysis to demonstrate that the complement pathway is upregulated in the retina 2 days after ONC [72], an observation that was repeated in our study. The immune system might also play an important role in the pathogenesis of axonal injury.

## Conclusion

We used RNA-seq technology to investigate the entire retinal transcriptome profile in the early stages of post-axonal injury. A pathway analysis of DEGs indicated that cell death and the survival response were induced at an early stage after ONC. ER stress was the main response in axonal injury, inducing many other pathways (i.e., RGC marker down regulation, the antioxidative response, the immune response, and axon regeneration). Our transcriptomic approach to this investigation, which relied on RNA-seq, was a powerful and effective method, and allowed us to obtain a global view of gene expression changes in the retina after axonal injury. We believe that our study has provided new insights into the molecular mechanisms underlying axonal damage, and may help in research aimed at the discovery of new biomarkers and therapeutic targets for a variety of ocular diseases.

## Materials and Methods

### Animals

C57BL/6 mice (male, 12-week-old; SLC, Hamamatsu, Japan) were used in this study. The surgical procedures were performed under deep anesthesia with intramuscular administration of a mixture of ketamine (100 mg/kg) and xylazine (9 mg/kg). All animals were maintained and handled in accordance with the guidelines of the ARVO Statement for the Use of Animals in Ophthalmic and Vision Research and the guidelines from the declaration of Helsinki and the Guiding Principles in the Care and Use of Animals. All experimental procedures described in the present study were approved by the Ethics Committee for Animal Experiments at Tohoku University Graduate School of Medicine, and were performed according to the National Institutes of Health guidelines for the care and use of laboratory animals.

### Induction of axonal injury in mice

ONC was used to induce axonal injury as previously described [10]. Briefly, the optic nerve was exposed, crushed 2 mm posterior to the globe with fine forceps for 10 seconds, and released. A fundus examination was used to confirm the appearance of normal blood circulation, and antibiotic ointment was applied. The operation was similar in the sham group, but after exposure, the optic nerve was not crushed.

### RNA extraction

Two days after surgery, the retinas of the mice were extracted and immediately immersed in an RNA stabilization reagent (RNase later sample and assay technology; Qiagen, Valencia, CA). The retinas were then homogenized in Qiazol (Qiagen) with a pestle homogenizer, and total RNA was extracted from the homogenized mixture with a miRNeasy mini kit (Qiagen). The resulting 48 individual samples (24 from the ONC group and 24 from the control group) were then assessed with a spectrophotometer to determine their total RNA concentration (NanoDrop 2000c, Thermo Scientific).

### RNA sequencing

Thirty-six samples of purified RNA (18 from the ONC and 18 from the control group) were used for this analysis. In each group, fixed quantities of RNA were taken from six samples and combined into a single sample, in order to minimize the influence of individual variations in the mice. This process yielded three combined samples from both the ONC and control groups. The quality of these six combined RNA samples was then assessed with an Agilent 2100 Bioanalyzer (Agilent Technologies, Palo Alto, CA). The triplicated ONC and control samples used for the RNA-seq analysis had RNA integrity numbers (RIN) ranging from 7.8 to 8.2 (Table S1). The cDNA library of each sample was prepared with Illumina Tru-Seq RNA Sample Prep Kits (Illumina, San Diego, CA) for 100 bp paired-end reads, according to the manufacturer’s instructions. Each of the six cDNA libraries was indexed for multiplexing. These six indexed libraries were sequenced on one lane of the Illumina Hiseq2000 device.

Data were recorded in the FASTQ format and then imported to CLC Genomics Workbench (version 6.0.1) (CLC Bio, Aarhus, Denmark) for analysis [18], [19]. All sequence reads were mapped to the reference genome (NCBI37/mm9) with the RNA-seq mapping algorithm included in CLC Genomics Workbench. The maximum number of mismatches allowed for the mapping was set at 2. To estimate gene expression levels, we calculated RPKM with CLC Genomics Workbench, as defined by Mortazavi et al. [20], and then analyzed differentially expressed genes (DEGs) in the control and ONC samples. All sequence data have been deposited in the Gene Expression Omnibus under the accession number GSE55228.

### Quantitative RT-PCR

Twelve samples of purified RNA (6 from the ONC and 6 from the control group) were used for quantitative RT-PCR. Total RNA (200 ng per sample) from the samples was first reverse-transcribed into cDNA using SuperScript III (Invitrogen Life Technologies, Carlsbad, CA). Quantitative RT-PCR was then performed with a 7500 Fast Real-Time PCR System (Applied Biosystems, Foster City, CA) as previously described, with minor modifications [73]. For each 20 μl reaction the following were used: 10 μl TaqMan Fast Universal PCR Master Mix (Applied Biosystems, Foster City, CA), 1 μl Taqman probe, 1 μl template DNA, and 8 μl DEPC water. Each sample was run in duplicate in each assay. For a relative comparison of gene expression, we analyzed the results of the real-time PCR data with the comparative Ct method (2^− ΔΔCT^), normalized to *Gapdh*, an endogenous control. All Taqman probes used for these reactions are listed in Table S5.

### Pathway analysis

Pathway and global functional analyses were performed with IPA software [36], [74], [75]. The DEG datasets were uploaded to the IPA application and mapped to IPKB. Each gene identifier was then mapped to its corresponding IPKB. Networks of these genes were generated based on their connectivity. The significance of the association between the datasets and biofunctions were measured using a ratio of number of genes from the dataset that map to the pathway divided by the total number of genes in that pathway. An upstream regulator analysis was performed to compare DEGs in the datasets to those known to be regulated by a given upstream regulator. Based on the concordance between them, an activation score was assigned, showing whether a potential transcriptional regulator was in an “activated” (z score ≥ 2), “inhibited” (z score ≤ −2), or uncertain state.

### Statistical analysis

RNA-seq data were analyzed and RPKM was calculated with CLC Genomics Workbench [76]. A threshold RPKM value of 0.3 has been reported to balance the numbers of false positives and false negatives [21], [77]. We therefore excluded genes that did not have RPKM > 0.3 in at least one group. This yielded 13160 genes, which we then used in the differential expression analysis. *P*-values were calculated with the Student’s t-test and were adjusted for multiplicity with the Bioconductor package qvalue [78], [79]. This software allows for selecting statistically significant genes while controlling the estimated false discovery rate (FDR). FDR < 0.1 with |FC| > 1.5 was considered statistically significant in the RNA-seq analysis. RT-PCR data were analyzed with the Welch’s t-test. Statistical analysis of the RNA-seq and RT-PCR data was performed with R software (version 3.0.1) [22]. The significance of the pathway analysis was calculated with Fisher’s exact test in the IPA application. If the *P*-values for RT-PCR and IPA were less than 0.05, the result was considered statistically significant.

## Supporting Information

Table S1 RNA integrity numbers and summary of sequence statistics.
(XLSX)

Table S2 Detailed mapping statistics.
(XLSX)

Table S3 List of DEGs after ONC.
(XLSX)

Table S4 List of DEGs belonging to cell death and survival after ONC.
(XLSX)

Table S5 List of Taqman probes used in this study.
(DOCX)
