# Supplementary material for: RNA Sequence Reveals Mouse Retinal Transcriptome Changes Early after Axonal Injury
Source: PLoS One. 2014 Mar 27;9(3):e93258. doi: 10.1371/journal.pone.0093258 (PMC3968129; doi:10.1371/journal.pone.0093258)
Supplement: Table S5 — List of Taqman probes used in this study. (DOCX) [file pone.0093258.s005.docx]

**Table S5. List of Taqman probes used in this study.**

| Gene symbol | Gene Accession |
| --- | --- |
| *Sprr1a* | Mm01962902_s1 |
| *Mmp12* | Mm00500554_m1 |
| *Sox11* | Mm01281943_s1 |
| *Atf3* | Mm00476032_m1 |
| *Tnfrsf12a* | Mm01302476_g1 |
| *Hmox1* | Mm00516007_m1 |
| *Plat* | Mm00476931_m19 |
| *Egr1* | Mm00656724_m1 |
| *Atf5* | Mm04179654_m1 |
| *Ddit3* | Mm01135937_g1 |
| *Jun* | Mm00495062_s1 |
| *Pou4f2* | Mm00454754_s1 |
| *Nefh* | Mm01191456_m1 |
| *Pou4f1* | Mm02343791_m1 |
| *Gapdh* | Mm999999115_g1 |
